# Loss of Conserved rRNA Modifications in the Peptidyl Transferase Center Leads to Diminished Protein Synthesis and Cell Growth in Budding Yeast

**DOI:** 10.3390/ijms25105194

**Published:** 2024-05-10

**Authors:** Margus Leppik, Liisa Pomerants, Anett Põldes, Piret Mihkelson, Jaanus Remme, Tiina Tamm

**Affiliations:** Institute of Molecular and Cell Biology, University of Tartu, 51010 Tartu, Estonia; margus.leppik@ut.ee (M.L.); liisa.pomerants@ut.ee (L.P.); anett.poldes@kliinikum.ee (A.P.); piret.mihkelson@ut.ee (P.M.); jaanus.remme@ut.ee (J.R.)

**Keywords:** budding yeast, ribosome, rRNA modifications, pseudouridine, ribose 2′-O-methylation, methyltransferase, Nop2, Spb1, snoRNA, translational fidelity

## Abstract

Ribosomal RNAs (rRNAs) are extensively modified during the transcription and subsequent maturation. Three types of modifications, 2′-O-methylation of ribose moiety, pseudouridylation, and base modifications, are introduced either by a snoRNA-driven mechanism or by stand-alone enzymes. Modified nucleotides are clustered at the functionally important sites, including peptidyl transferase center (PTC). Therefore, it has been hypothesised that the modified nucleotides play an important role in ensuring the functionality of the ribosome. In this study, we demonstrate that seven 25S rRNA modifications, including four evolutionarily conserved modifications, in the proximity of PTC can be simultaneously depleted without loss of cell viability. Yeast mutants lacking three snoRNA genes (*snR34*, *snR52,* and *snR65*) and/or expressing enzymatically inactive variants of *spb1(D52A/E679K)* and *nop2(C424A/C478A)* were constructed. The results show that rRNA modifications in PTC contribute collectively to efficient translation in eukaryotic cells. The deficiency of seven modified nucleotides in 25S rRNA resulted in reduced cell growth, cold sensitivity, decreased translation levels, and hyperaccurate translation, as indicated by the reduced missense and nonsense suppression. The modification m^5^C2870 is crucial in the absence of the other six modified nucleotides. Thus, the pattern of rRNA-modified nucleotides around the PTC is essential for optimal ribosomal translational activity and translational fidelity.

## 1. Introduction

Three classes of RNA molecules (mRNA, rRNA, and tRNA) work together to achieve fast and accurate protein synthesis. All these RNAs undergo post-transcriptional chemical modification. Transfer RNAs (tRNAs) are extensively modified; on average, about 17% of total residues are modified [1]. Ribosomal RNAs (rRNAs) contain up to 4% of modified nucleotides [2]. Even messenger RNAs (mRNAs) have been found to contain modified nucleotides in eukaryotes, and knowledge about the role of mRNA modifications in the control of translation is steadily increasing [3,4]. It has been proposed that RNA modifications play a role in RNA dynamics, e.g., stabilising and fine-tuning its structure and function [5]. Nevertheless, the biological role of the majority of RNA modifications in translation remains enigmatic.

In rRNAs, the most abundant modifications are the 2′-O-methylations of ribose sugars and isomerisation of uridines to pseudouridines (Ψ). In addition, different types of base modifications have been characterised [6]. Although the overall structure of the core ribosome has been well-conserved during evolution, the architectural complexity of the ribosome has increased, including the abundance of modified nucleotides in rRNA [7]. In bacterial *E. coli* ribosome, rRNAs contain 36 modified nucleotides, whereas more than 100 modified nucleotides have been found in the yeast *S. cerevisiae* ribosome and over 200 in the human ribosome [2,7,8]. The higher number of modified nucleotides is mainly attributed to the increased ribose 2′-O-methylation and pseudouridylation [7].

From bacteria to humans, not only has the number of modified nucleotides in rRNAs increased, but, also, more diverse mechanisms for site-specific modification have been implemented. In bacteria, all the modifications are synthesised by specific enzymes known as stand-alone modification enzymes [9]. In archaea and eukaryotes, the stand-alone enzymes are responsible for introducing the base modifications. The 2′-O-methylations and Ψ are mostly guided by protein complexes containing small nucleolar RNAs (snoRNAs), and these catalytically functional complexes are called box C/D and box H/ACA snoRNPs, respectively [10,11]. Thus, more diverse mechanisms correlate with the higher number of modified nucleotides in the rRNAs. Moreover, the snoRNA-dependent modification mechanism is more cost-effective, explaining the increased number of Ψ and 2′-O methylation modifications.

Modified rRNA nucleotides tend to cluster in functionally important regions such as tRNA binding sites, the peptidyl transferase center (PTC), the decoding center, the peptide exit tunnel, and intersubunit bridges [12]. Only nine modified positions in rRNA from *E. coli* to *H. sapiens* are universally conserved, of which three are located in the ribosomal small subunit (SSU) and six in the large subunit (LSU) (MODOMICS, [13]). Of the six LSU conserved modified nucleotides, four are located in the PTC region [14], emphasising their functional importance.

In the budding yeast *S. cerevisiae*, the universally conserved modifications around the PTC are introduced by a snoRNA-driven mechanism and two stand-alone enzymes. snR34 directs box H/ACA snoRNP to catalyse the modification Ψ2826 (Ψ2457 in *E. coli*) [15]. snR52 and snR67 direct box C/D snoRNPs to modify U2921 (Um2552 in *E. coli*) and G2619 (Gm2251 in *E. coli*), respectively [16]. Modification m^5^C2870 (ho^5^C2501 in *E. coli*) is catalysed by the stand-alone enzyme Nop2 [13]. Interestingly, Um2921 is catalysed by two separate mechanisms. In the absence of snR52, this position is methylated by the stand-alone enzyme Spb1 [17]. In wild-type cells, Spb1 catalyses the formation of Gm2922 [18]. It is not clear whether such a backup system is required because the modification Um2921 is biologically crucial or whether it is an enzymatic by-product due to the catalytic pocket architecture of Spb1, as previously speculated for the pseudouridine synthase RluD [19].

Although the deletion of individual snoRNA genes has little or no effect on yeast growth, the catalytic subunits of the snoRNPs, Cbf5 and Nop1, are essential for life [20,21,22]. Intriguingly, the expression of the catalytically inactive Cbf5, *cbf5(D95A*), can rescue the lethal phenotype of *CBF5* deletion in yeast and human cells [23,24]. This results in global rRNA pseudouridylation defects affecting ribosome–ligand interactions and leading to altered translation fidelity [24].

Even though Nop2 and Spb1 modify only two nucleotides in the vicinity of PTC in wild-type cells, the deletion of genes encoding these enzymes is lethal [22,25]. Studies in budding yeast have shown that both enzymes are required for pre-rRNA processing, and, thus, are critical for LSU assembly [18,26]. The lethal phenotype can be rescued by the expression of Nop2 and Spb1 methyltransferase-deficient variants *nop2(C478A)* and *spb1(D52A)*, respectively [13,17,27,28]. These mutants were viable but displayed a severe growth defect due to impaired LSU biogenesis [13,17,18]. Notably, the growth defect caused by the methyltransferase-deficient *nop2(C478A)* allele was suppressed by introducing a second cysteine mutation in the Nop2 active site [27]. Cells expressing the *nop2(C424A/C478A*) allele were viable and exhibited a growth rate similar to the wild-type. Similarly, the severe growth defect of *spb1(D52A)* can be compensated by a suppression mutation E769K at the C-terminal domain of Spb1 [29]. It was shown that the catalytic activity of Spb1 is associated with Nog2, a small GTPase involved in the maturation of the 60S subunit [29]. Unmethylated G2922 causes the premature activation of Nog2 GTPase activity, which prevents Nog2 binding to 60S intermediates during the late step of ribosome biogenesis. The suppression mutation E769K does not restore the Spb1 methyltransferase activity but restores the Nog2 binding to 60S intermediates to facilitate maturation [29].

It is known that rRNA modifications are redundant, making it difficult to define the relationship of the rRNA modification landscape to ribosome function and/or assembly. The absence of several modifications simultaneously may shed light on the function of a single modified rRNA nucleotide. In this study, we investigated the role of seven rRNA modifications in the proximity of PTC, including four evolutionarily conserved modifications, in ensuring ribosome functionality. To achieve this, we combined deletions of three snRNA genes (*snR34*, *snR52,* and *snR67*) with Nop2 and Spb1 methyltransferase-deficient alleles, which do not significantly affect cell growth. The analysis of the mutants demonstrated that rRNA modifications in PTC contribute in concert to efficient translation in eukaryotic cells. The absence of seven modified nucleotides in LSU resulted in reduced translational levels and altered translational fidelity. Although these rRNA modifications are dispensable for cell growth, they play an important role in ribosome translational activity and translational fidelity.

## 2. Results

**Figure 1:**
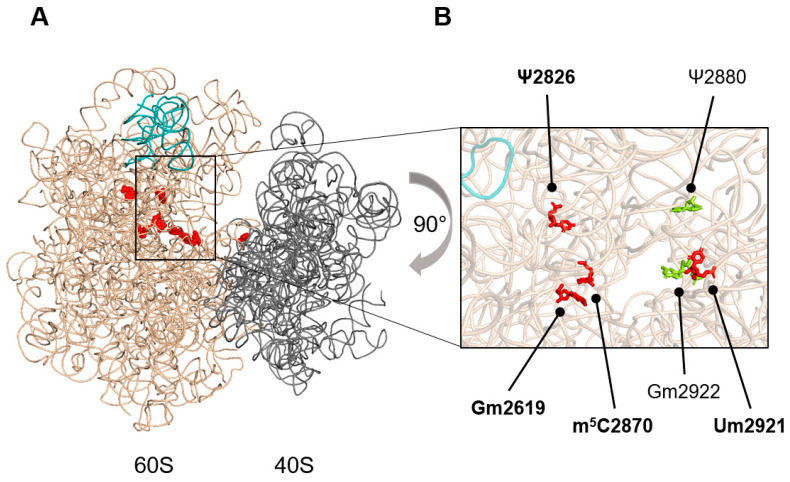
Structure of the *S. cerevisiae* 80S ribosome with the rRNA modifications investigated in this study. (**A**) Overview of the *S. cerevisiae* 80S ribosome. The rRNA of 60S and 40S subunits, respectively, are shown in wheat and light grey. The 5S rRNA is shown in teal. The location of the eight rRNA modifications absent in 3ΔsnR nop2* spb1* mutant is indicated (red). (**B**) Zoomed-in view of 60S subunit peptidyl transferase center region. The rRNA modifications that are affected in 3ΔsnR nop2* spb1* mutant are shown. Evolutionarily conserved modifications are shown in red, non-conserved modifications in light green. The ribosome structure was generated by PyMOL [30] using the coordinates form [31] PDB ID: 4V88.

Budding yeast 25S rRNA harbours four evolutionarily conserved modifications close to PTC (Figure 1). To evaluate how the absence of these rRNA modifications affects ribosome functionality, we constructed yeast strains lacking one to seven LSU rRNA modifications (Table 1, Supplementary Figure S1).

**Figure 2:**
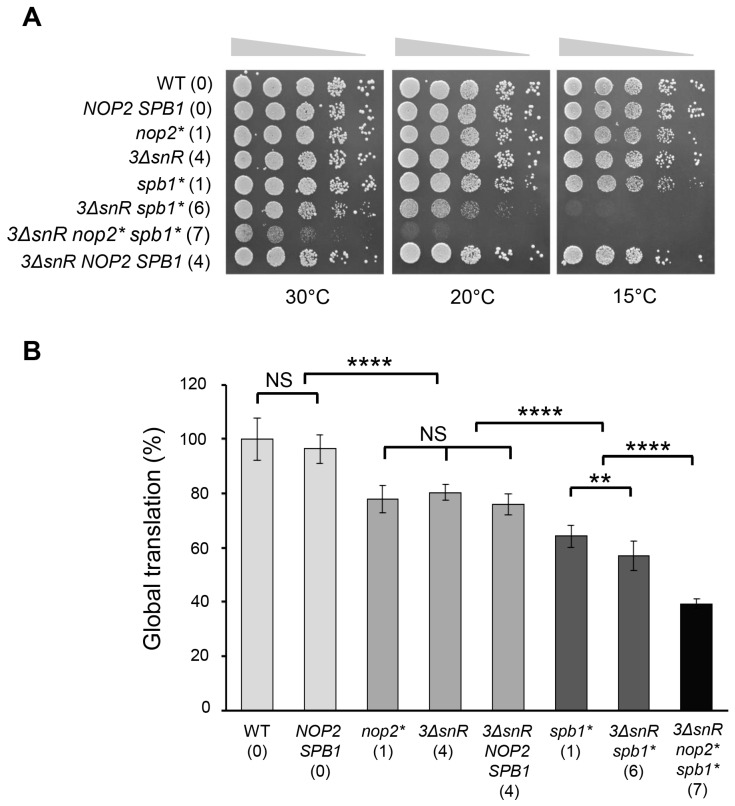
Phenotypic characterisation of rRNA modification mutants. (**A**) Serial dilutions of wild-type (WT) and indicated mutant strains were spotted onto rich medium. Cells were grown at the indicated temperatures for 2–8 days. (**B**) Analysis of levels of global translation. The incorporation of radioactive isotope-labelled amino acids into newly synthesised polypeptides was measured in exponentially growing cells at 30 °C. Samples were TCA-precipitated and the incorporation of radioactive label was measured over time. The obtained values of DPM were normalised to the optical density values of the sample, and the slope was calculated. The average slope values (mean ± SD) from at least nine biological replicates are plotted and are shown relative to the wild-type. The numbers in parentheses next to the strain names indicate the number of missing modifications in the 25S rRNA. Statistical significance was determined by the unpaired two-sample Student’s *t*-test (** *p* < 0.01; **** *p* < 0.0001; NS, not significant).

Nop2 catalyses the methylation of C2870 at C5 [13]. In mutant nop2*, a methyltransferase-deficient *nop2(C424A/C478A)* was expressed in a *nop2Δ* background, resulting in the loss of m^5^C2870 (Supplementary Figure S1) as previously reported [13,27]. In mutant *3ΔsnR*, the genes encoding snoRNAs snR34, snR52, and snR67 were deleted. These snoRNAs guide the modification of five rRNA residues in LSU, including conserved Gm2619 and Ψ2826, and one rRNA residue in SSU (Figure 1, Supplementary Figure S1). The methylation of U2921 is ensured by two mechanisms. This modification is mainly guided by snR52 [16,17]. In the case of *snR52* deletion, the residue U2921 is modified by conserved methyltransferase Spb1 (Supplementary Figure S1) [17,18]. To abolish U2921 methylation, the deletion of *snR52* must be combined with the expression of a methyltransferase-deficient variant of Spb1. For this, we used the *spb1(D52A/E679K)* allele, where the mutation in the active site is accompanied by a suppression mutation in the Spb1 C-terminal domain [29]. The expression of *spb1(D52A/E679K)* in the *sbp1Δ* background (mutant spb1*) rescues the growth defect reported for the catalytically deficient *spb1(D52A)* mutant [17,18,29] (Figure 2A). In mutant 3ΔsnR spb1*, the deletion of three snoRNA genes was combined with a methyltransferase-deficient variant of Spb1, resulting in the abolition of six LSU rRNA modifications. In mutant 3ΔsnR nop2* spb1*, the deletion of three snoRNA genes was combined with the expression of methyltransferase-deficient variants of Nop2 and Spb1, resulting in the loss of all seven rRNA modifications in the LSU (Table 1). The successful construction of such a viable mutant demonstrates that ribosomes lacking eight rRNA modifications, including the evolutionarily conserved Gm2619, Ψ2826, m^5^C2870, and Um2921, retain functionality.

### 2.1. The Absence of PTC rRNA Modifications Leads to Growth Defects and Changes in Global Protein Translation

To investigate in detail the growth characteristics of the modification-deficient strains, we performed a serial dilution spot-test on rich medium at different temperatures and measured the generation times in rich medium at 30 °C (Figure 2A, Supplementary Figure S2, Table 2). To evaluate the translation efficiency in vivo, we determined the levels of global translation (Figure 2B). For that, the incorporation of radioactive isotope-labelled amino acids in newly synthesised polypeptides was measured in exponentially growing yeast cells.

The loss of one or four rRNA modifications in LSU has a modest effect on ribosomes. Mutants nop2* and *3ΔsnR* exhibited growth similar to wild-type at all temperatures tested (Figure 2A, Table 2). In addition, the levels of translation in both strains were reduced by 1.3 times compared to wild-type (Figure 2B). Based on these results, we suggest that m^5^C2870 alone does not play a major role in ribosome functioning.

*SPB1* is an essential gene, but the expression of methyltransferase-deficient *spb1* allele rescues the lethal phenotype [17,22,28]. The spb1* mutant displayed a 1.4-fold increase in generation time and a 1.6-fold decrease in translation compared to the wild-type (Figure 2, Table 2). Although the phenotypes observed for the *spb1(D52A/E679K)* allele demonstrate a significant difference from the wild-type, these are much weaker than the phenotypes observed for the previously reported *spb1(D52A)* allele [17,18]. The loss of six or seven rRNA modifications in LSU (mutants 3ΔsnR spb1*, and 3ΔsnR nop2* spb1*) resulted in impaired growth and reduced translation (Figure 2, Table 2). Both mutants showed reduced growth at 30 °C and cold sensitivity at 20 °C compared to wild-type or spb1*. The generation time of 3ΔsnR spb1* mutant was 1.9 times longer compared to wild-type. The generation time of 3ΔsnR nop2* spb1* mutant was 2.1 times longer than that of spb1* cells and 3 times longer than in wild-type cells (Table 2). A significant 2.6-fold reduction in translation compared to wild-type was observed in this mutant (Figure 2B). It is important to note that the expression of wild-type *NOP2* and *SPB1* in the *3ΔsnR nop2Δ sbp1Δ* background restored growth and translation to the levels observed in *3ΔsnR* cells. Thus, the absence of seven rRNA modifications leads to a considerable reduction in the level of global translation. Modifications Ψ2826, Um2921, Gm2619, m5C2870, Ψ2880, Um2724, and Gm2922 play a role in ribosome functioning.

Taken together, these results indicate that the rRNA modification m^5^C2870 becomes crucial in the absence of six other modifications. The mutant 3ΔsnR nop2* spb1* lacking all seven modified nucleotides displayed a longer generation time and a reduced level of translation compared to cells lacking one to six rRNA modifications in LSU. Using the *spb1(D52A/E679K)* allele allowed us to evaluate the role of Um2921 and Gm2922 in ribosome functioning. Our results demonstrate that Gm2922 alone has an intermediate effect on ribosome functioning. However, the absence of Um2921 and Gm2922, in combination with four additional rRNA modifications, leads to a significant reduction in cell growth and levels of global translation compared to strains in which these modifications were present. This highlights the importance of Um2921 and Gm2922 in the case where other rRNA modifications are not present.

### 2.2. rRNA Modifications Contribute to Translational Fidelity

To determine the role of these rRNA modifications in translational fidelity, we used an in vivo bicistronic dual-luciferase reporter system [32,33]. The control reporter encodes the *Renilla*-firefly luciferase fusion protein. In the reporter plasmids, the translation of firefly luciferase is dependent on a defect in translational fidelity. The use of different reporter plasmids allowed the measurement of −1 programmed ribosomal frameshifting (−1 PRF), +1 PRF, the readthrough of UAA or UAG termination codons (i.e., nonsense suppression), and the suppression of the serine-encoding AGC codon by a near-cognate aa-tRNA that normally decodes the AGA arginine codon (i.e., misincorporation).

**Figure 3:**
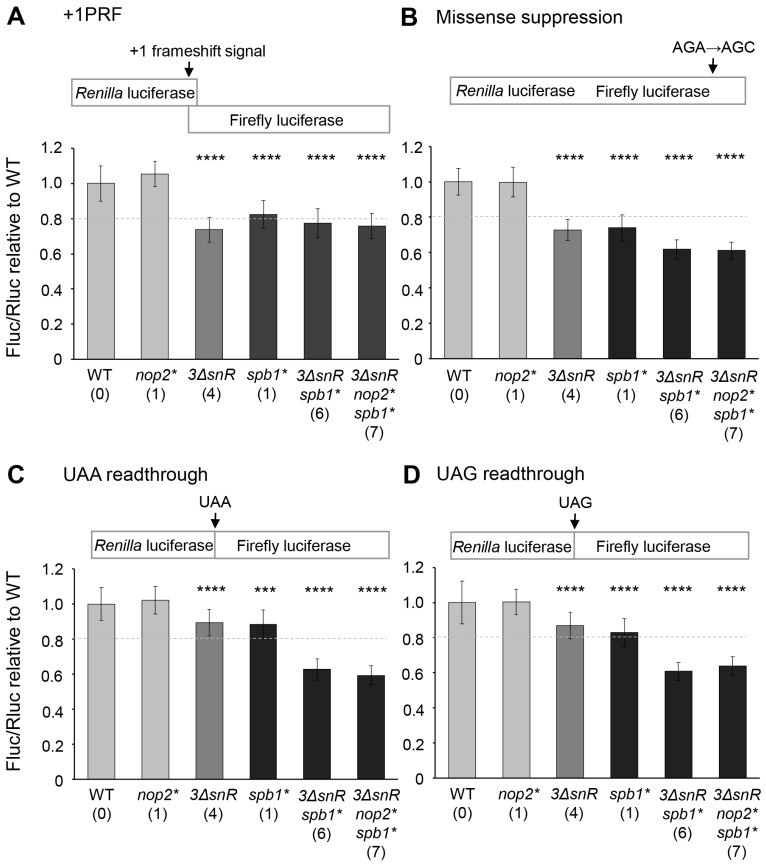
Translational fidelity of rRNA modification mutants. Wild-type cells (WT) and indicated mutants were transformed with dual-luciferase reporters and control plasmid. The activities of both luciferases were measured. The ratio of firefly luciferase to *Renilla* luciferase activities was normalised to the control plasmid and is shown relative to the wild-type. The numbers in parentheses next to the strain names indicate the number of missing modifications in the 25S rRNA. (**A**) +1 PRF was measured using the Ty1 frameshift signal. (**B**) The rate of missense suppression was evaluated by the incorporation of an arginine near-cognate amino acid instead of a cognate serine at the catalytic codon 218 in the firefly luciferase. (**C**,**D**) Termination codon readthrough was measured using reporters containing in-frame UAA and UAG termination codons between the *Renilla* and firefly luciferase coding regions. Each dataset represents the average (mean ± SD) of at least 15 biological replicates. Asterisks above columns indicate statistically significant changes compared to wild-type as determined by the unpaired two-sample Student’s *t*-test (*** *p* < 0.001; **** *p* < 0.0001). A 1.2-fold difference in wild-type was counted as biologically different, as described earlier [34].

All three mutants carrying deletions of snoRNA genes (3ΔsnR, 3ΔsnR spb1*, and 3ΔsnR nop2* spb1*) showed a significant decrease in +1 PRF (1.3-fold and 1.4-fold lower than wild-type) and misreading of AGC codon (1.4-fold and 1.6-fold lower than wild-type) (Figure 3, Supplementary Table S1). Moreover, a reduced level of arginine misincorporation at the AGC codon in the spb1* mutant compared to the wild-type was measured. A substantial effect was observed in the recognition of termination codons. Mutants 3ΔsnR spb1* and 3ΔsnR nop2* spb1* became hyperaccurate at UAA and UAG. The readthrough of termination codons was 1.6 times lower in these mutants than in wild-type cells. The mutant nop2* showed no changes in translation fidelity (Figure 3, Supplementary Figure S3, Supplementary Table S1). None of the mutants tested had a significant effect on −1 PRF (less than 1.2-fold difference compared to wild-type) (Supplementary Figure S3, Supplementary Table S1).

Altogether, the loss of rRNA modifications had different impacts on the translation fidelity. The absence of a conserved m^5^C2870 showed no effect on the translation accuracy, even when it was absent in combination with other modifications tested. The loss of four modifications, including Gm2619 and Ψ2826, had a significant effect on the +1 PRF and the misreading of the near-cognate codon. In the absence of two modifications, Um2921 and Gm2922, the translation became more accurate, as evidenced by the decrease in the termination codon readthrough.

### 2.3. Loss of rRNA Modifications Lowers the Number of Actively Translating Ribosomes

Protein translation levels were globally affected in mutants lacking multiple rRNA modifications. To characterise the translation in more detail, we performed the ribosome-polysome profile analysis and calculated the polysome/monosome ratios (P/M).

**Figure 4:**
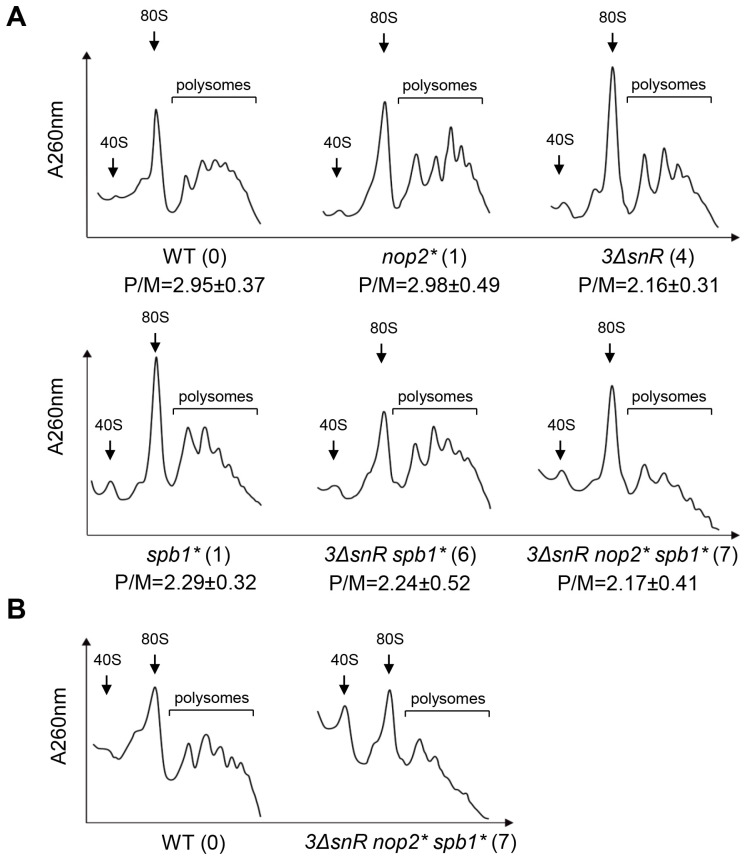
Ribosome-polysome profiles of rRNA modification mutants. Analysis of ribosome-polysome profiles of wild-type (WT) and indicated mutants by sedimentation in sucrose density gradient. (**A**) Cells were grown in rich medium at 30 °C to mid-exponential phase and analysed. (**B**) Cells were cultured at 30 °C to mid-exponential phase and shifted to 20 °C for 2 h (WT) or 4 h (mutant 3ΔsnR nop2* spb1*) and analysed. The whole-cell extracts were prepared from cycloheximide-treated cells and analysed in 7–47% sucrose gradients. The absorbance at 260 nm (A260 nm) was recorded. Sedimentation is from left to right. The peaks of free 40S ribosomal subunits, monosomes (80S), and polysomes are indicated. To determine the P/M ratio, the areas under the monosome and polysome peaks were quantified by ImageJ, and the ratio was calculated. The average (mean ± SD) ratios of at least three biological replicates are indicated. The numbers in parentheses next to the strain names indicate the number of missing modifications in the 25S rRNA.

Wild-type cells showed a high fraction of polysomes (P/M ratio ~3) and a low number of free subunits at 30 °C (Figure 4A). No significant changes in the P/M ratio were observed in extracts from methyltransferase-deficient nop2* mutant cells compared to wild-type (Figure 4A). In contrast, in extracts from mutants lacking three snoRNA genes and/or expressing the *spb1(D52A/E679K)* variant in the *sbp1Δ* background, the P/M ratios were reduced 1.4-fold and a considerable increase of free 40S subunits was observed compared to wild-type extracts. The 3ΔsnR nop2* spb1* cell extract displayed a similar phenotype at 30 °C, e.g., low levels of polysomes and accumulation of free subunits.

Previous studies established the role of Spb1 in ribosome biogenesis [17,18]. The expression of the *spb1(D52A)* variant in the *sbp1Δ* background caused the appearance of stalled preinitiation complexes called “halfmers” at 30 °C. This phenotype was more pronounced when the *spb1(D52A)* allele was combined with the deletion of the *snR52*, which led to the loss of Um2921 and Gm2922. The accumulation of “halfmers” is associated with a shortage of 60S subunits due to impaired 60S biogenesis. Ribosome biogenesis defects are more pronounced at lower temperatures. To test whether the expression of the *spb1(D52A/E679K)* variant rescues the ribosome biogenesis defect, we shifted cultures grown at 30 °C to 20 °C for one generation time, and then carried out the ribosome-polysome profile analysis (Figure 4B). No “halfmer” formation was registered in cell extracts of the 3ΔsnR nop2* spb1* mutant at 20 °C, indicating that the absence of seven modifications does not result in a strong defect in LSU biogenesis. These results are consistent with the cell growth analysis, where the expression of *spb1(D52A/E679K)* restored cell growth similar to wild-type.

In conclusion, our results indicate that the loss of rRNA modifications causes the reduction of polysomes and increase in free 40S subunits (Figure 4), which is in agreement with the reduced levels of global translation.

The absence of rRNA modifications in LSU may interfere with the assembly of ribosomal proteins during ribosome biogenesis, therefore reducing the amount of functional 80S ribosomes in the cells. This would explain the decreased growth rate, overall translation levels, and P/M ratio in the 3ΔsnR nop2* spb1* mutant. To test this possibility, we used quantitative mass spectrometry analysis for a more in-depth analysis of the composition of mutant 80S ribosomes. “Heavy” wild-type 80S ribosomes were obtained via stable isotope labelling by amino acids in cell culture (SILAC). Both mutant and “heavy” wild-type 80S ribosomes were separated in sucrose density gradients, mixed at an equimolar ratio, and the abundance of ribosomal proteins was determined by HPLC-MS/MS. No significant changes in the stoichiometry of ribosomal proteins were detected in 80S ribosomes of 3ΔsnR nop2* spb1* mutant compared to wild-type (Supplementary Figure S4).

In summary, the analysis of the ribosomal proteins revealed that ribosome composition and the stoichiometry of ribosomal proteins in 80S ribosomes of 3ΔsnR nop2* spb1* mutant are similar to those of wild-type ribosomes. It is likely that ribosome maturation in mutants carrying the *spb1(D52A/E679K)* allele may occur in a similar manner to wild-type. Thus, the observed phenotypes, e.g., prolonged generation time, reduced translation, and changes in translational fidelity, are probably caused by the absence of rRNA modifications in LSU.

## 3. Discussion

In this study, we aimed to elucidate the function of conserved modified nucleotides in the PTC region. We constructed budding yeast mutants lacking one to seven modifications (Ψ2826, Um2921, Gm2619, m5C2870, Ψ2880, Um2724, and Gm2922) in 25S rRNA. These modifications are directed by the essential stand-alone modification enzymes Nop2 and Spb1, and by snoRNPs harbouring snoRNAs snR34, snR52, and snR67. The deficiency of seven modified nucleotides in PTC resulted in more severe phenotypes, e.g., the longer generation time (Table 2), cold sensitivity and reduction of global translation level (Figure 2), decreased population of actively translating ribosomes (Figure 4), and increased translational fidelity (Figure 3), than those observed in mutants lacking up to six modifications. A similar synergistic effect was also reported when the importance of multiple Ψs in the PTC was examined [35]. The deletion of one to five snoRNA genes yielded strains lacking one to six Ψs. In the absence of all six Ψs in the PTC, the impairment of the cells was more severe than that detected with the loss of five or fewer Ψs.

The methyltransferase Nop2 is responsible for the synthesis of m^5^C2870 in 25S rRNA. Nop2 contains two conserved cysteine residues, C424 and C478, which are predicted to be important for SAM binding [27]. To investigate the impact of the absence of m^5^C2870, we exploited a methyltransferase-defective *nop2(C424A/C478A)* double mutant allele. The constructed nop2* strain was indistinguishable from the wild-type in terms of the growth rate, translational fidelity, and abundance of actively translating ribosomes. However, the importance of m^5^C2870 modification becomes evident in the absence of other modifications Ψ2826, Um2921, Gm2619, Ψ2880 Um2724, and Gm2922 in 25S rRNA. Compared with mutant 3ΔsnR spb1*, strain 3ΔsnR nop2* spb1* exhibited a significant 1.5-fold reduced level of global translation, a 1.4-fold longer generation time, and a more pronounced cold-sensitive phenotype (Figure 2, Table 2). Notably, these severe phenotypes depend on the absence of the Nop2-directed m^5^C2870 modification, demonstrating that this modification contributes to translation in co-operation with other modifications. A similar cooperation of modifications has also been reported in the absence of several Ψs in the budding yeast PTC region [35].

The methyltransferase Spb1 is responsible for the synthesis of Gm2922 in the A-loop of the PTC region [18]. When Um2921 is unmodified due to the absence of *SNR52*, this nucleotide is modified by Spb1 [17]. In *E. coli*, the corresponding modification Um2552 is synthesised by methyltransferase RlmE, which was first identified as a heat shock protein [36]. Like Spb1 in yeast, RlmE is one of the few modification enzymes whose deletion causes severe growth and ribosome biogenesis defects in bacteria [37]. The defects in growth and ribosome biogenesis resulting from the deletion of *rlmE* can be modulated by small GTPases. Specifically, the overexpression of two small GTPases, ObgE and EngA, compensates for the phenotypic defect of *rlmE* deletion [38], suggesting that RlmE has a second function in addition to its methyltransferase activity. It has also been shown that the 2′-O methyl group of U2552 (U2921 in yeast) is necessary in order to facilitate interdomain interactions during the late step of LSU assembly [39], making this modification highly important for ribosomes. Furthermore, the effect of a lack of RlmE is severe in the absence of nine other 23S rRNA modification enzymes [40].

We constructed an enzymatically inactive allele of Spb1 methyltransferase *(D52A/E679K)* to investigate the role of Um2921 and Gm2922 modifications. The observed phenotypes were significantly milder than previously reported for the strain *spb1(D52A)* [18], making the allele *spb1(D52A/E679K)* more suitable for studying the effects of Spb1-dependent modifications. Spb1-directed modifications Um2921 and Gm2922 become important in the absence of 25S rRNA modifications Gm2619, Ψ2880, Um2724, and Ψ2826. Compared with *3ΔsnR*, strain 3ΔsnR spb1* exhibited a 1.4-fold reduced level of global translation, cold sensitivity (Figure 2), 1.6 times longer generation time (Table 2), 1.2 times reduced missense suppression, and 1.4 times reduced stop codon readthrough (Figure 3, Supplementary Table S1). Interestingly, the decreased level of global translation and growth cold sensitivity do not correlate with the actively translating ribosome population (Figure 4). Hyperaccurate ribosomes are known to translate at a slow rate [41], which may explain the low translation level of modification-deficient strains. The results demonstrating a reduced stop codon readthrough are in good agreement with the previously reported phenotype of the *spb1(D52A)/ΔsnR52* strain [34]. Interestingly, a previous study has shown that the absence of Um2921 and Gm2922 increases the frequency of −1 PRF, and +1 PRF, and the misincorporation of amino acids [34]. In contrast, our strain 3ΔsnR spb1* showed a higher fidelity and mRNA reading frame maintenance compared to wild-type (Figure 3, Supplementary Table S1), suggesting that modifications Ψ2826, Gm2619, Ψ2880, and Um2724 play an important role in codon recognition and reading frame maintenance. The simulation of interactions between aa-tRNA and rRNA during the accommodation step involves 25S rRNA helixes 89, 90, and 92 (A-loop), which guide the tRNA CCA end toward the peptidyl transferase center [42]. The modifications studied in this work (Ψ2826, Ψ2880, m^5^C2870, Um2921, and Gm2922) are located in this region. The cumulative effect of the modification deletions leads to a speculation that the modifications stimulate the conformational changes needed for the aa-tRNA accommodation into the A-site. Thus, our results are consistent with the proposal that the modifications have a synergic effect on translation [35].

In this study, we demonstrate that severe phenotypic defects due to the expression of methyltransferase-deficient modification enzymes can be overcome using the Nop2 and Spb1 alleles *nop2(C424A/C478A)* and *spb1(D52A/E679*. The use of these alleles was instrumental in overcoming the limitations arising from the expression of the *nop2C478A* and *spb1D52A* alleles, which hampered the investigation of the function of evolutionarily conserved rRNA modifications in the PTC region. We were able to eliminate all the conserved modifications from the PTC region without the loss of *S. cerevisiae* viability. It can be concluded that the presence of seven modifications (Ψ2826, Um2921, Gm2619, m5C2870, Ψ2880, Um2724, and Gm2922) in 25S rRNA optimises the PTC region for optimal translation accuracy. Moreover, our results indicate that PTC modifications have a cumulative effect on translation, as recently shown in *E. coli* [40]. Although modifications have a fine-tuning effect on the rRNA structure and dynamics, we show that different modifications contribute to different aspects of translation. It is likely that modifications are needed for the PTC dynamics, leading to a slower accommodation, which potentially increases the time for the discrimination of aa-tRNA during the proofreading step. Our results are consistent with the idea that modifications modulate the rRNA structure for optimal translation.

## 4. Materials and Methods

### 4.1. Yeast Strains and Media

All *S. cerevisiae* strains used in this study were isogenic derivatives of strain S288C [43]. All strains used are listed in Supplementary Table S2.

Cells were grown in rich medium (YPD, 1% Bacto yeast extract, 2% Bacto peptone, and 2% glucose) or in synthetic complete medium (0.67% Bacto yeast nitrogen medium without amino acids, and 2% glucose) supplemented with the appropriate amino acids and bases [44]. Agar (2%) was added to solid media. When required, the following concentrations of antibiotics were used: 200 mg/l geneticin, 300 mg/l hygromycin B, and 100 mg/l nourseothricin.

Strains *ΔsnR34*, *ΔsnR52,* and *ΔsnR67* were generated by one-step PCR-based gene disruption [45] of the *YNCL0048W*, *YNCE0020C*, and *YNCE002W* genes, respectively. All oligonucleotide primers used for gene disruption are listed in Supplementary Table S3. Deletion cassette containing *loxP-kanMX4-loxP* were PCR-amplified using appropriate primer pairs and transformed into TYSC309 and TYSC310 strains. Yeast cells were transformed using lithium acetate/polyethylene glycol method [46]. For marker cassette rescue, cells were transformed with the Cre recombinase expression plasmid pSH47 [47]. The expression of the Cre recombinase was induced in galactose-containing media. Cells were plated on YPD plates and the loss of the marker gene was verified by PCR. The strain TYSC736 was created by multiple rounds of crossings of the obtained strains. The detailed mating scheme is shown in Supplementary Figure S5A.

Strains TYSC763, TYSC775, TYSC821, TYSC833, TYSC844, TYSC854, TYSC893, and TYSC898 were constructed by a one-step PCR-based gene disruption method [45]. To generate strains TYSC821, TYSC833, TYSC844, and TYSC854, a deletion cassette containing *natMX6* [48] was PCR-amplified and used to disrupt *NOP2* gene in diploids TYSC628 or TYSC745. The resulting heterozygous diploids were transformed with *pRS315-NOP2* or pRS315-nop2* plasmids, and sporulated, and tetrads were dissected. To construct strains TYSC763, TYSC775, TYSC893, and TYSC898, the PCR-amplified *hphMX6* cassette [48] was transformed into strains TYSC628 or TYSC745 to yield a *SPB1/Δspb1* diploid and *SPB1/Δspb1 ΔsnR34/ΔsnR34 ΔsnR52/ΔsnR52 ΔsnR67/ΔsnR67* diploid, respectively. Plasmids *pRS314-SPB1* or pRS315-spb1* were transformed into a heterozygous diploid followed by sporulation and tetrad dissection.

Construction of all other mutants is summarised in Supplementary Figure S5B,C. The genotypes of the obtained colonies were analysed by PCR.

### 4.2. Plasmids

All plasmids used in the study are listed in Supplementary Table S4. Oligonucleotide primers used for plasmid construction are listed in Supplementary Table S3.

The *NOP2* coding region with its 5’ upstream (288 bp) and 3’ downstream (410 bp) regions was amplified by PCR from genomic DNA and cloned between *PstI* and *XhoI* restriction sites into the *pRS315* vector, resulting in plasmid *pRS315-NOP2*. To generate *pRS315-nop2(C424A/C478A)* plasmid, the site-directed mutagenesis was performed by overlap extension PCR to introduce C424A and C478A substitutions into *pRS315-NOP2*.

For construction of *pRS314-SPB1* plasmid, the *SPB1* coding region with 483 bp upstream and 263 bp downstream sequences was amplified from genomic DNA by PCR and cloned between *XhoI* and *SacI* restriction sites into the *pRS314* vector. To generate *pRS314-spb1(D52A/E679K)* plasmid, the site directed mutagenesis was performed to introduce these substitutions into *pRS314-SPB1*.

### 4.3. Generation Time Measurements

To analyse cell growth, the overnight grown cultures were diluted into fresh YPD medium. The aliquots of 150 μL cultures were incubated in 96-well plate shaker-reader Polarstar Omega at 30 °C. The OD_600_ was measured at 15 min intervals. The generation times for at least eight biological replicates with three parallel cultures were calculated from the exponential growth phase and presented as mean values with standard deviations. Statistical significance was determined by the unpaired two-sample Student’s *t*-test.

### 4.4. Temperature Sensitivity Assays

Yeast strains were grown in YPD media at 30 °C to mid-exponential phase. The cultures were serially diluted and spotted on YPD plates and incubated at 16, 20, 25, 30, and 36 °C for 2–8 days.

### 4.5. Analysis of Global Level of Translation

For analysis of global level of translation, cells were grown in synthetic complete medium at 30 °C in the presence of C14-labeled amino acids. The analysis was performed as previously described [49,50]. Cultures in exponential growth phase were sampled every 15 min for 2 hr. In contrast, samples from the cultures of 3ΔsnR spb1* (strain TYSC898) and 3ΔsnR nop2* spb1* (strain TYSC906) were taken every 30 min for 4 hr. The DPM (disintegration per minute) values of incorporated amino acids were normalised with the optical density values (OD_600_) of the sample and plotted against the corresponding time points. The slopes of the obtained curves were calculated. The average and standard deviations for at least nine biological replicates were calculated. The statistical significance was determined by the unpaired two-sample Student’s *t*-test.

### 4.6. Ribosome-Polysome Profile Analysis

The preparation of cell extracts and ribosome-polysome profile analysis were carried out as described earlier [49,51], with modifications. Notably, 150 mL of yeast cells were grown until OD_600_ of 0.9–1.1 and treated with cycloheximide at a final concentration of 100 μg/mL 15 min before harvesting. Cells were washed twice in ice-cold breaking buffer (10 mM Tris-HCl (pH 7.5), 100 mM NaCl, and 30 mM MgCl_2_) supplemented with 100 µg/mL cycloheximide, 2 mM DTT, and 0.5 mM PMSF. After disrupting cells in breaking buffer with 0.5 mm glass beads in Precellys 24 homogeniser, obtained extract was centrifuged at 16,060× *g* for 3 × 5 min. Thirty A_260_ units of cell extract were layered onto 7–47% sucrose gradient supplemented with 2 mM DTT and 0.5 mM PMSF, and centrifuged at 4 °C in SW28 rotor (Beckman Coulter, Brea, CA, USA) at ω^2^t = 1.8 × 10^11^. Sedimented ribosome particles in gradients were monitored at 260 nm. For polysome/monosome ratio quantification, the areas under the monosome (80S) and polysome peaks were quantified by ImageJ and corresponding ratios were calculated. The average and standard deviations of polysome/monosome ratios for at least three biological replicates were calculated.

### 4.7. Analysis of Translational Fidelity

The dual-luciferase reporter plasmids (Supplementary Table S4) were used to quantitatively measure −1 programmed ribosomal frameshifting, +1 programmed ribosomal frameshifting, UAA and UAG codon readthrough, and suppression of an AGC serine codon in place of an AGA arginine codon. Cells transformed with either in-frame *Renilla*-firefly luciferase control or reporter plasmids were grown in synthetic complete medium to exponential growth phase. The cells were collected by centrifugation and resuspended in 1× passive lysis buffer (Promega Corporation, Madison, WI, USA). Luminescence measurements were performed using the Dual-Luciferase Reporter Assay System (Promega) with Infinite M200 Pro Multimode Microplate Reader (Tecan Group Ltd., Männedorf, Switzerland). For each plasmid, at least 15 biological replicates were analysed. For data analysis, the ratio of firefly to *Renilla* luciferase activity was calculated for each plasmid. The translational fidelity was calculated by dividing the firefly/*Renilla* ratios determined from cells expressing the reporter plasmids by the firefly/*Renilla* ratio determined from cells expressing the in-frame control plasmid. The statistical significance was determined by the unpaired two-sample Student’s *t*-test.

## Figures and Tables

**Table 1:** Analysed mutants.

Strain Name	Genotype ^1^	Missing Modification(s) ^2^	Number of Missing LSU Modification(s)
*NOP2 SPB1*	*Δnop2 + NOP2* *Δspb1 + SPB1*	-	0
nop2*	*Δnop2 + nop2(C424A/C478A)*	**m^5^C2870**	1
*3* *ΔsnR*	*ΔsnR34 ΔsnR52 ΔsnR67*	**Gm2619** Um2724 **Ψ2826** Ψ2880	4
*3* *ΔsnR NOP2 SPB1*	*ΔsnR34 ΔsnR52 ΔsnR67* *Δnop2 + NOP2* *Δspb1 + SPB1*	**Gm2619** Um2724 **Ψ2826** Ψ2880	4
spb1*	*Δspb1 + spb1(D52A/E679K)*	Gm2922	1
*3* ΔsnR spb1*	*ΔsnR34 ΔsnR52 ΔsnR67* *Δspb1 + spb1(D52A/E679K)*	**Gm2619** Um2724 **Ψ2826** Ψ2880 **Um2921** ^3^ Gm2922	6
*3* ΔsnR nop2* spb1*	*ΔsnR34 ΔsnR52 ΔsnR67* *Δnop2 + nop2(C424A/C478A)* *Δspb1 + spb1(D52A/E679K)*	**Gm2619** Um2724 **Ψ2826** Ψ2880 **Um2921** ^3^ Gm2922 **m^5^C2870**	7

^1^ All strains are isogenic derivatives of strain S288C—the detailed genotypes of the strains are provided in Supplementary Table S2; ^2^ Only modifications in the 25S rRNA are shown—evolutionarily conserved modifications are in bold; ^3^ Um2921 is introduced by two systems: snoRNP-dependent (*snR52*) and site-specific methyltransferase Spb1.

**Table 2:** Growth of analysed mutants.

Strain Name ^a^	Number of Missing LSU Modification(s) ^b^	Generation Time (min) ^c^	Fold Change (Mutant to WT)
WT	0	^1^ 91.0 ± 6.2	1.0
*NOP2 SPB1*	0	^1^ 92.0 ± 6.6	1.0
nop2*	1	^1^ 94.6 ± 3.4	1.0
*3* *ΔsnR*	4	^2^ 110.2 ± 5.8	1.2
*3* *ΔsnR NOP2 SPB1*	4	^2^ 110.0 ± 6.1	1.2
spb1*	1	^3^ 127.3 ± 4.9	1.4
*3* ΔsnR spb1*	6	^4^ 175.0 ± 9.4	1.9
*3* ΔsnR nop2* spb1*	7	^5^ 272.6 ± 15.1	3.0

^a^ Cells were grown in rich medium (YPD) at 30 °C; ^b^ Only the number of missing modifications in the 25S rRNA is indicated; ^c^ Generation times were calculated from at least eight biological replicates in triplicate; means are shown with standard deviations. Numbers (1–5) indicate statistically homogenous groups according to unpaired two-sample Student’s *t*-test (*p* < 0.01). The same numbers denote no statistically significant difference.

## Data Availability

The data presented in this study are available upon request from the corresponding author. The mass spectrometry proteomics data have been deposited to the ProteomeXchange Consortium via the PRIDE partner repository with the dataset identifier PXD051211.

## Supplementary Materials

The following supporting information can be downloaded at: https://www.mdpi.com/article/10.3390/ijms25105194/s1, References [19,32,33,34,43,47,49,50,52,53,54,55,56] are cited in the supplementary materials.
