# Supplementary material for: Loss of Conserved rRNA Modifications in the Peptidyl Transferase Center Leads to Diminished Protein Synthesis and Cell Growth in Budding Yeast
Source: Int J Mol Sci. 2024 May 10;25(10):5194. doi: 10.3390/ijms25105194 (PMC11121408; doi:10.3390/ijms25105194)
Supplement: Supplementary file 1 [file ijms-25-05194-s001.zip › ijms-2975450-supplementary.pdf]

Loss of Conserved rRNA Modifications in the Peptidyl Transferase Centre Leads to Diminished Protein Synthesis and Cell Growth in Budding Yeast

Margus Leppik, Liisa Pomerants, Anett Põldes, Piret Mihkelson, Jaanus Remme and Tiina Tamm

SUPPLEMENTARY MATERIAL

Supplementary Figures S1-S5

Supplementary Table S1-S4

Supplementary Methods

Supplementary References

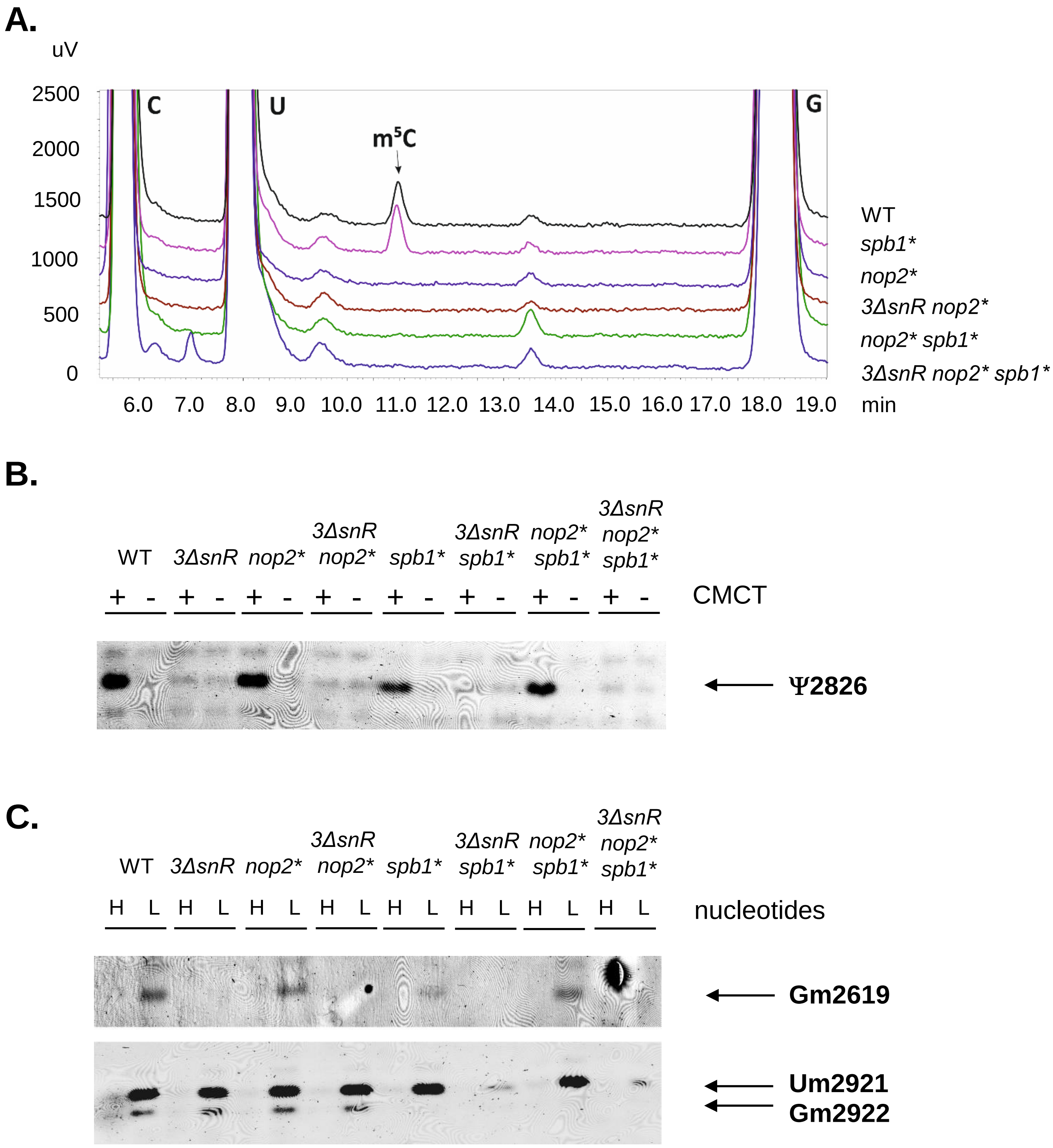

Supplementary Figure S1. Analysis of 25S rRNA evolutionarily conserved modifications in constructed mutants.
(A) RP-HPLC analysis of 25S rRNA fragment. Nucleoside composition of RNase H digested fragment corresponding to region 2685-2892 of 25S rRNA from wild-type and indicated mutants. The peak corresponding to m^5C is shown.
(B) CMCT-alkali primer extension analysis to map Ψ2826. "+" lines correspond to CMCT treated samples and "–" lanes untreated control samples.
(C) High and low nucleotide concentration primer extension analysis to map Gm2619, Um2921 and Gm2922 rRNA modifications. "H" corresponds to samples containing 1 mM dNTP in primer extension samples and "L" correspond to 0.1 mM dNTP sample.
(B-C) 7% PAAG electrophoresis was used to separate DNA fragments. Oligonucleotides containing 5'-FAM were used for visualization of primer extension products.

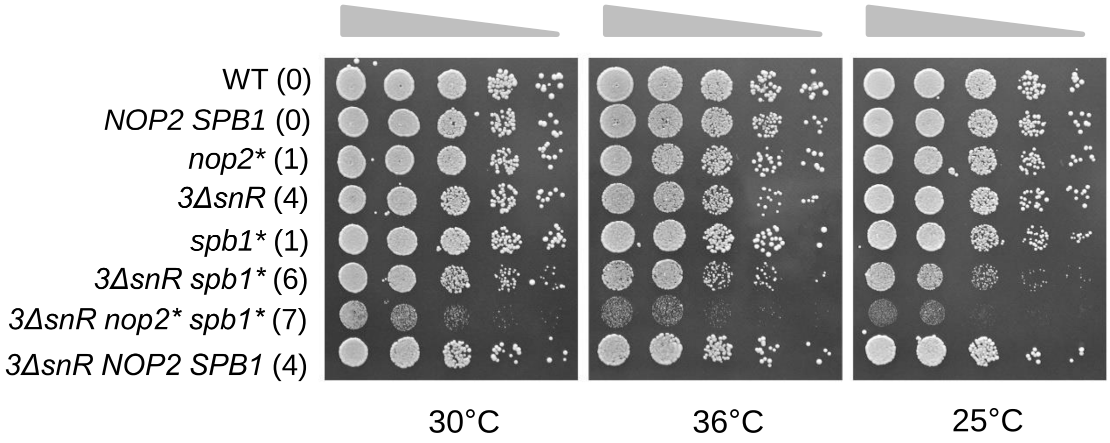

Supplementary Figure S2 (relates to Figure 2). Phenotypic characterisation of rRNA modification mutants.

Serial dilutions of wild-type (WT) and indicated mutant strains were spotted onto rich medium. Cells were grown at the indicated temperatures for 2-3 days. The numbers in parentheses next to the strain names indicate the number of missing modifications in the 25S rRNA.

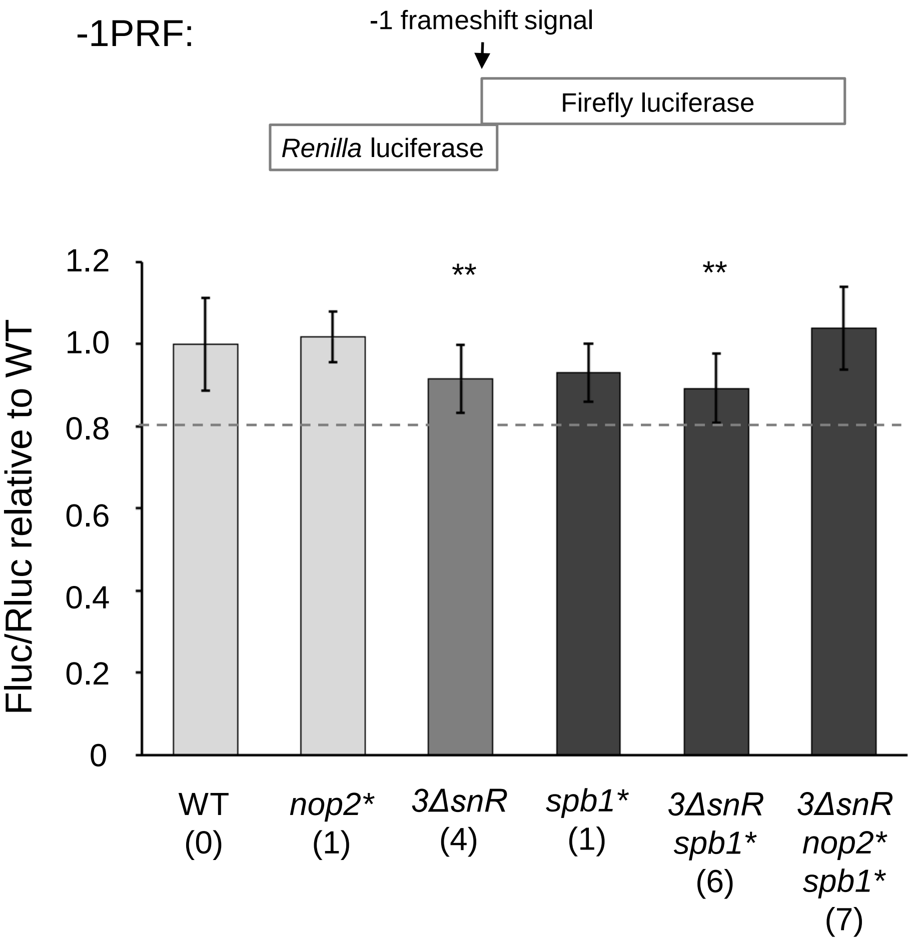

Supplementary Figure S3 (relates to Figure 3). Translational fidelity of rRNA modification mutants.

Wild-type cells (WT) and indicated mutants were transformed with dual-luciferase reporter and control plasmid. The activities of both luciferases were measured. The ratio of firefly luciferase to *Renilla* luciferase activities was normalised to the control plasmid and is shown relative to the wild type. -1 PRF was measured using L-A frameshift signal. Each dataset represents the average (mean ± SD) of at least 15 biological replicates. The numbers in parentheses next to the strain names indicate the number of missing modifications in the 25S rRNA. Asterisks above columns indicate statistically significant changes compared to wild-type as determined by the unpaired two-sample Student's *t*-test (***P*<0.01). A 1.2-fold difference in wild type was counted as biologically different as described earlier [13].

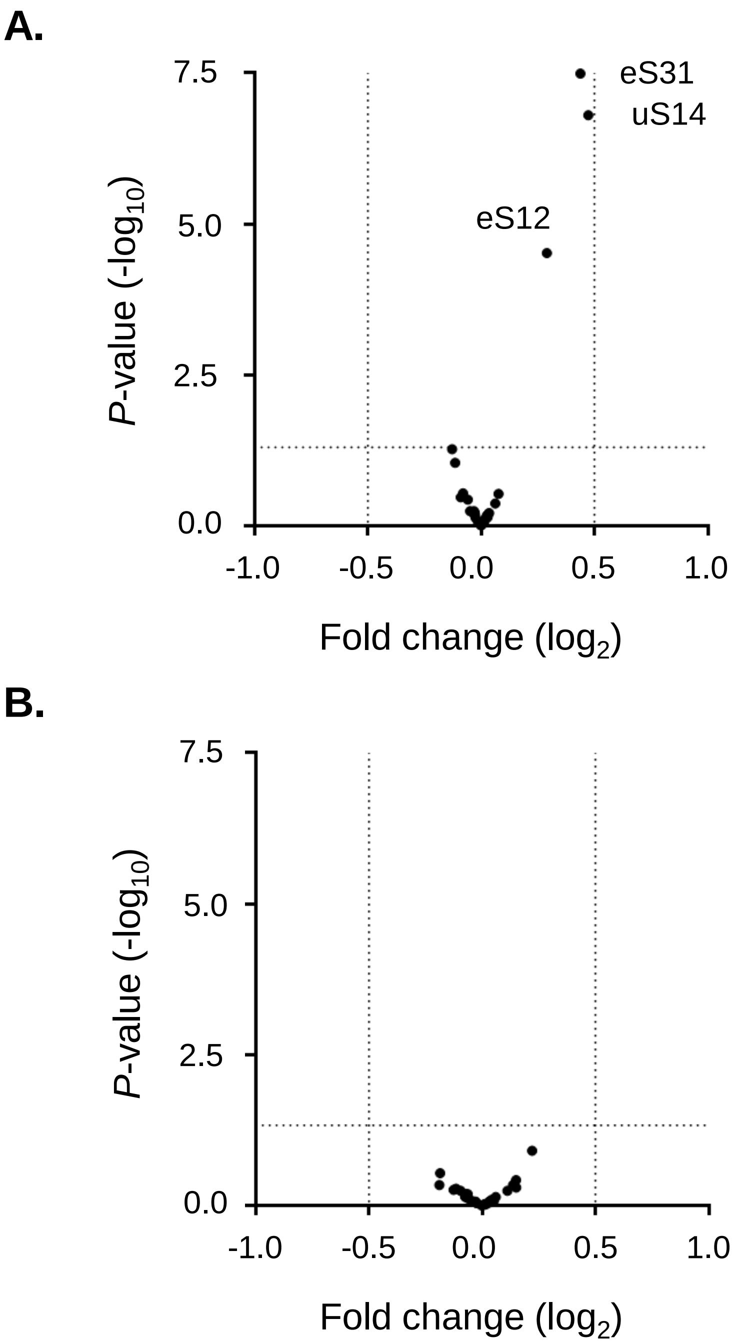

Supplementary Figure S4. Mass spectrometric analysis of 80S ribosomes from wild-type and *3ΔsnR nop2* spb1** mutant.

(A) Stochiometry of SSU proteins in 80S ribosomes. **(B)** Stochimetry of LSU proteins in 80S ribosomes.

Wild-type and mutant cells were grown in rich YPD medium (e.g. “light” medium). Wild-type cells for reference were grown in “heavy” synthetic minimal medium, L-lysine and L-arginine in the medium were exchanged to [$^{13}C_6/^{15}N_2$] L-lysine and [$^{13}C_6/^{15}N_4$] L-arginine. All 80S ribosomes were isolated by separation in sucrose density gradients. “Light” mutant or wild-type 80S ribosomes were mixed at an equimolar ration with “heavy” wild-type reference 80S ribosomes, and “heavy”/”light” ratios of ribosomal proteins were determined by MS/MS.

Volcano plots show the changes in stoichiometry of ribosomal proteins in ribosomes of mutant versus wild-type. Horizontal dashed line indicates threshold *P*-value of 0.05 ($-\log_{10} = 1.33$). Vertical dashed lines indicate threshold fold change values 1.41 ($\log_2 = 0.5$) and -1.41 ($\log_2 = -0.5$).

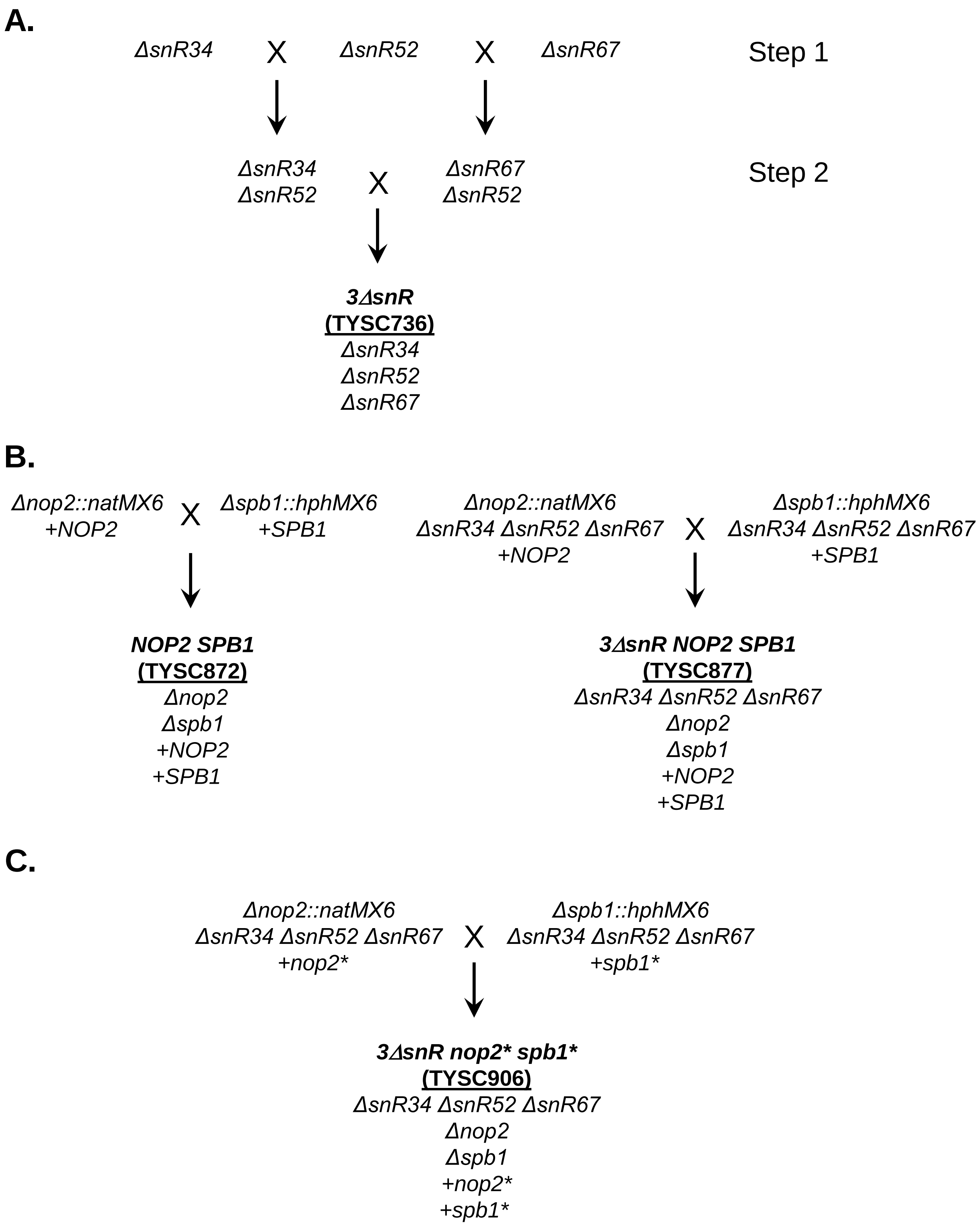

Supplementary Figure S5. Diagrams illustrating the strategy for strain construction.
(A) Strategy for construction of *3ΔsnR* strain (TYSC736)
(B) Strategy for construction of *NOP2 SPB1* (TYSC872) and *3ΔsnR NOP2 SPB1* (TYSC877) strains.
(C) Strategy for construction of *3ΔsnR nop2* spb1** (TYSC906) strain.

Supplementary Table S1. Summary of translational fidelity phenotypes (relates to Figure 3 and Supplementary Figure S3).

Strain		WT	*nop2**	*3ΔsnR*	*spb1**	*3ΔsnR spb1**	*3ΔsnR nop2* spb1**
**Programmed frameshifting**							
**-1 PRF**	Efficiency (%)	7.3±0.8	7.4±0.5	6.7±0.6	6.8±0.5	6.5±0.6	7.6±0.7
	Fold WT		1.02	0.91	0.93	0.89	1.04
	p-values[1]		5.46E-01	5.80E-03	2.56E-02	3.19E-03	2.56E-01
**+1 PRF**	Efficiency (%)	11.2±1.1	11.8±0.8	8.3±0.8	9.2±0.9	8.7±0.9	8.5±0.8
	Fold WT		1.05	0.74	0.82	0.77	0.76
	p-values[1]		5.14E-02	2.55E-13	3.97E-08	5.88E-10	2.19E-11
**Misincorporation**							
**Near-cognate**	Efficiency (%)	0.087 ±0.007	0.087 ±0.007	0.063 ±0.005	0.065 ±0.006	0.054 ±0.005	0.054 ±0.004
	Fold WT		1.00	0.73	0.74	0.62	0.61
	p-values[1]		9.49E-01	1.20E-15	3.37E-14	5.98E-21	3.75E-18
**Nonsense suppression**							
**UAA**	Efficiency (%)	0.36 ±0.03	0.37 ±0.03	0.32 ±0.03	0.32 ±0.03	0.22 ±0.02	0.21 ±0.02
	Fold WT		1.00	0.89	0.89	0.63	0.60
	p-values[1]		3.95E-01	7.45E-05	2.41E-04	2.16E-18	9.82E-21
**UAG**	Efficiency (%)	0.38 ±0.05	0.38 ±0.03	0.33 ±0.03	0.31 ±0.03	0.23 ±0.02	0.24 ±0.02
	Fold WT		1.00	0.87	0.83	0.61	0.64
	p-values[1]		8.88E-01	6.56E-05	2.48E-05	1.75E-15	1.24E-14

[1] Statistical significance was determined by the unpaired two-sample Student's *t*-test.

Supplementary Table S2. Yeast strains used in this study.

Strain	Strain name	Genotype	Source
TYSC309	WT	*MAT**a** ura3-52 leu2Δ1 his3Δ200 trp1Δ36 Δarg4 Δlys1*	Lab collection
TYSC310		*MATα ura3-52 leu2Δ1 his3Δ200 trp1Δ36 Δarg4 Δlys1*	Lab collection
TYSC628		*MAT**a/**MATα ura3-52/ura3-52 leu2Δ1/leu2Δ1 his3Δ200/his3Δ200 trp1Δ36/trp1Δ36 Δarg4/Δarg4 Δlys1/Δlys1*	Lab collection
TYSC736	*3ΔsnR*	*MAT**a** ura3-52 leu2Δ1 his3Δ200 trp1Δ36 Δarg4 Δlys1 ΔsnR34 ΔsnR52 ΔsnR67*	This study
TYSC745		*MAT**a/**MATα ura3-52/ura3-52 leu2Δ1/leu2Δ1 his3Δ200/his3Δ200 trp1Δ36/trp1Δ36 Δarg4/Δarg4 Δlys1/Δlys1 ΔsnR34/ΔsnR34 ΔsnR52/ΔsnR52 ΔsnR67/ΔsnR67*	Lab collection
TYSC763	*SPB1*	*MAT**a** ura3-52 leu2Δ1 his3Δ200 trp1Δ36 Δarg4 Δlys1 Δspb1::hphMX6 [pRS314-SPB1]*	This study
TYSC775	*3ΔsnR SPB1*	*MAT**a** ura3-52 leu2Δ1 his3Δ200 trp1Δ36 Δarg4 Δlys1 ΔsnR34 ΔsnR52 ΔsnR67 Δspb1::hphMX6 [pRS314-SPB1]*	This study
TYSC821	*NOP2*	*MAT**a** ura3-52 leu2Δ1 his3Δ200 trp1Δ36 Δarg4 Δlys1 Δnop2::natMX6 [pRS315-NOP2]*	This study
TYSC833	*nop2**	*MAT**a** ura3-52 leu2Δ1 his3Δ200 trp1Δ36 Δarg4 Δlys1 Δnop2::natMX6 [pRS315-nop2(C424A, C478A)]*	This study
TYSC844	*3ΔsnR NOP2*	*MAT**a** ura3-52 leu2Δ1 his3Δ200 trp1Δ36 Δarg4 Δlys1 ΔsnR34 ΔsnR52 ΔsnR67 Δnop2::natMX6 [pRS315-NOP2]*	This study
TYSC854	*3ΔsnR nop2**	*MAT**a** ura3-52 leu2Δ1 his3Δ200 trp1Δ36 Δarg4 Δlys1 ΔsnR34 ΔsnR52 ΔsnR67 Δnop2::natMX6 [pRS315-nop2(C424A, C478A)]*	This study

TYSC872	*NOP2 SPB1*	*MAT**a** ura3-52 leu2Δ1 his3Δ200 trp1Δ36 Δarg4 Δlys1 Δnop2::natMX6 Δspb1::hphMX6 [pRS314-SPB1] [pRS315-NOP2]*	This study
TYSC877	*3ΔsnR NOP2 SPB1*	*MAT**a** ura3-52 leu2Δ1 his3Δ200 trp1Δ36 Δarg4 Δlys1 ΔsnR34 ΔsnR52 ΔsnR67 Δnop2::natMX6 Δspb1::hphMX6 [pRS314-SPB1] [pRS315-NOP2]*	This study
TYSC893	*spb1**	*MAT**a** ura3-52 leu2Δ1 his3Δ200 trp1Δ36 Δarg4 Δlys1 Δspb1::hphMX6 [pRS314-spb1(D52A, E679K)]*	This study
TYSC898	*3ΔsnR spb1**	*MAT**a** ura3-52 leu2Δ1 his3Δ200 trp1Δ36 Δarg4 Δlys1 ΔsnR34 ΔsnR52 ΔsnR67 Δspb1::hphMX6 [pRS314-spb1(D52A, E679K)]*	This study
TYSC906	*3ΔsnR nop2* spb1**	*MAT**a** ura3-52 leu2Δ1 his3Δ200 trp1Δ36 Δarg4 Δlys1 ΔsnR34 ΔsnR52 ΔsnR67 Δnop2::natMX6 Δspb1::hphMX6 [pRS314-spb1(D52A, E679K)] [pRS315-nop2(C424A, C478A)]*	This study

Supplementary Table S3. Oligonucleotides used in this study.

Name	Sequence	Used for
snR34_DEL_S1	5’ CAGTCAACAACAATTTTGAGTAT ATTTACCACATTTTTTCTGATTTTTT TTTAAGAGGGTCGTACGCTGCAG GTCGAC 3’	*snR34* deletion
snR34_DEL_S2	5’ AATATTGAAACTGATCTTCAAGA CTTTTTACCTGCAAGAGACAGTAC GTAAATATATGAGATCGATGAATT CGAGCTCG 3’	*snR34* deletion
snR52_DEL_S1	5’ TGAAAGTTGGTGCGCATGTTTC GGCGTTCGAAACTTCTCCGCAGT GAAAGATAAATGATCCGTACGCTG CAGGTCGAC 3’	*snR52* deletion
snR52_DEL_S2	5’ CTAGACAGAAGTTTGCGTTCCA TACTGTCAGAGGTGGCATTTTACA TAACAATAGTGACAATCGATGAAT TCGAGCTCG 3’	*snR52* deletion
snR67_DEL_S1	5’ ATTGTTCCTTTAGAGATGATAAA GACAACTTACAAGTACAGTTTTTT GTTGGTATCTCATCGTACGCTGCA GGTCGAC 3’	*snR67* deletion
snR67_DEL_S2	5’ TTCTTGCATTAAATACATGTTCC TTGAGAAAACTTTTGATGTTTAATG TGTAAATATCTCATCGATGAATTC GAGCTCG 3’	*snR67* deletion
SPB1_DEL_S1	5’ CAATACTCATTCTGCTTCAGTTT GTAGTTAGATTTAACTCAATAGAG GTGATTGGCAAAACGTACGCTGCA GGTCGAC 3’	*SPB1* deletion
SPB1_DEL_S2	5’ CTTTCTTTTCCTTTATTTTCAATA TTATACAAGGGAATGGAAAAATAA TGCTCTTTGTTAATCGATGAATTC GAGCTCG 3’	*SPB1* deletion
NOP2_DEL_S1	5’ TTCTGGCGGAATTCCCCTTGTG TTGGCTAATATTAGAATTACATATA	*NOP2* deletion

	CATATAATAGGAACGTACGCTGCA GGTCGAC 3’	
NOP2_DEL_S2	5’ ACAGAGAAAGATTAGAGAGAGA AAACTATGCTAACATGATGCCACT ACGTTTGTGGGAACATCGATGAAT TCGAGCTCG 3’	*NOP2* deletion
SPB1_FORW_XhoI	5’ CAACCTCGAGGTCCAGCAATCC GGCGCAAAGAAGAC 3’	*pRS314-SPB1* construction
SPB1_REV_SacI	5’ CACCGAGCTCAATGGTACGTTA CTATGCTTACTCATC 3’	*pRS314-SPB1* construction
NOP2_FORW_PstI	5’ GAACCTGCAGGCAACAATGTGA GTAGGATCCAACGTG 3’	*pRS315-NOP2* construction
NOP2_REV_XhoI	5’ CAACCTCGAGACTGGAAGGGTA TGGCAGCTCTAGCAC 3’	*pRS315-NOP2* construction
SPB1_D52A_MUT_F	5’ TCGAAGGTTGTTATTGCTCTGTG TGCTGCTCCTGGTTCATGGTGTCA AG 3’	site-directed mutagenesis
SPB1_D52A_MUT_R	5’ CCAGGAGCAGCACACAGAGCAA TAACAACCTTCGATTTTTCTAAGAA GTGG 3’	site-directed mutagenesis
SPB1_E769K_MUT_F	5’ GATTAACGATGATTCAGACAAG ACAAAGAAGGACAAGGCTGAAGA AATTTCTAG 3’	site-directed mutagenesis
SPB1_E769K_MUT_R	5’ CTAGAAATTTCTTCAGCCTTGTC CTTCTTTGTCTTGTCTGAATCATCG TTAATC 3’	site-directed mutagenesis
NOP2_C424A_MUT_F	5’ CAGAATTTTACTGGATGCCCCA GCTTCCGGTACTGGTGTTATCGGT AAGG 3’	site-directed mutagenesis
NOP2_C424A_MUT_R	5 CTTACCGATAACACCAGTACCG GAAGCTGGGGCATCCAGTAAAATT CTG 3’	site-directed mutagenesis
NOP2_C478A_MUT_F	5 GTGTAATAGTATATTCGACAGCT TCTGTTGCAGTGGAAGAGGACGA AGC 3	site-directed mutagenesis
NOP2_C478A_MUT_R	5’ GCTTCGTCCTCTTCCACTGCAA CAGAAGCTGTCGAATATACTATTA CACC 3’	site-directed mutagenesis

Sc25S2964 (Gm2922/Um2921)	5’ CGACGGTCTAAACCCAGCTCAC 3’	primer extension
Sc25S2774 (Um2724)	5’ GGGACTAAAGGATCGATAGGCC 3’	primer extension
Sc25S2654 (Gm2619)	5’ GGACATCTGCGTTATCGTTTAAC AGATGTG 3’	primer extension
Sc25S2930 (PSI2880)	5’ TGGGTGAACAATCCAACGCTTA CC 3’	primer extension
Sc25S2863 (PSI2826)	5’ CAAAAAGCAATGTCGCTATGAA CGCTTGAC 3’	primer extension
Sc25S2918	5’ CCAACGCTTACCGAATTCTGCT TCGG 3’	25S rRNA fragmentation
Sc25S2685	5’ GGAGATTTCTGTTCTCCATGAG CCC 3’	25S rRNA fragmentation

Supplementary Table S4. Plasmids used in this study.

Plasmid	Description	Source, reference
pSH47	*P_{GAL1}-Cre / URA3 / CEN*	[1]
pRS314	*TRP1 / CEN*	[2]
pRS315	*LEU2 / CEN*	[2]
pYDL-control (pJD375)	*Rluc-Fluc / URA3 / CEN*	[3]
pYDL-LA (pJD376)	*Rluc-LA-Fluc / URA3 / CEN*	[3]
pYDL-Ty1 (pJD377)	*Rluc-Ty1-Fluc / URA3 / CEN*	[3]
pYDL-UAA (pJD431)	*Rluc-UAA-Fluc / URA3 / CEN*	[3]
pYDL-UAG (pJD432)	*Rluc-UAG-Fluc / URA3 / CEN*	[3]
pYDL-AGC_{218} (pJD643)	*Rluc-FlucAGC_{218} / URA3 / CEN*	[4]
pRS314-SPB1	*SPB1 / TRP1 / CEN*	This study
*pRS314-spb1**	*spb1(D52A, E679K) / TRP1 / CEN*	This study
pRS315-NOP2	*NOP2 / LEU2 / CEN*	This study
*pRS315-nop2**	*nop2(C424A, C478A) / LEU2 / CEN*	This study

SUPPLEMENTARY METHODS

Analysis of the modified nucleotides

To analyse the ribose methylation status of Gm2922, Gm2619, Um2724 and Um2921, primer extension analysis was performed as described [5]. Briefly, two separate primer extension reactions were performed with each primer, one reaction with high (1 mM) and the other reaction with low (0.1 mM) nucleotide concentration. Reverse transcriptase (Promega Corporation, Madison, WI, USA) and the 5' FAM (Fluorecein)-labelled primers listed in Supplementary Table S3 were used. The resulting DNA fragments were resolved in a 7% polyacrylamide-urea gel. Fluorescently labelled fragments were visualised with Typhoon Trio Imager (GE HealthCare, Chicago, Illinois, USA) and low nucleotide concentration specific polymerase stop signals were analysed.

Pseudouridines were determined by RNA-CMCT-alkali treatment and primer extension as previously described [6]. The primer extension reaction using reverse transcriptase (Promega Corporation, Madison, WI, USA) and the 5' FAM (Fluorecein)-labeled primers listed in Supplementary Table S3 were carried out. The resulting DNA fragments were resolved in a 7% polyacrylamide-urea gel. Fluorescently labelled fragments were visualised by Typhoon Trio Imager (GE HealthCare, Chicago, Illinois, USA) and CMCT-dependent polymerase stop signals were analysed.

To map the m^5C2870 modification, a fragment of 25S rRNA corresponding nucleotides 2686-2892 was excised by RNaseH using oligonucleotides complementary to nt 2661-2685 and 2893-2918 of *S. cerevisiae* rRNA. The primer hybridization, RNaseH cleavage reaction and RNA fragment purification was carried out as previously described [7]. rRNA P1 digestion and HPLC analysis was performed as described by [8].

LC-MS/MS analysis of 80S ribosomes

The purification of 80S ribosomes was carried out as described earlier [9, 10]. Wild-type (TYSC309) and *3ΔsnR nop2* spb1** (TYSC906) cells were grown in YPD medium. To label proteins with 'heavy' amino acids, wild-type (TYSC309) yeast cells were grown in synthetic minimal medium as described previously [9]. Cells were collected by low speed centrifugation, lysed and ribosomes were separated in 10%-30% sucrose density gradient. Collected 80S fractions were pelleted by ultracentrifugation. For mass spectrometric analysis 'light' and 'heavy' ribosomes were mixed in 1:1 ratio, the samples were TCA precipitated and acetone washed. Proteins were reduced for 1 hour at RT by adding 5 mM DTT and carbamidomethylated with 20 mM chloroacetamide for 1 hour at RT in the dark. Proteins were digested with endoproteinase Lys-C (FUIJIFILM Wako Chemicals U.S.A. Corporation, Richmond, VA, USA) at an enzyme to protein ratio 1:50 for 4 hours at RT. The urea concentration in the solutions was reduced by adding 4 volumes of 100 mM NH_3HCO_3 and peptides

were further digested with mass spectrometry grade trypsin (Sigma Aldrich, Saint Louis, MO, USA; enzyme to protein ratio 1:50) at RT for overnight. Enzymes were inactivated by addition of TFA to a final concentration of 1%.

Two independent biological replicates for each strain were analysed. Peptides were desalted on self-made reverse-phase C_{18} stop and go extraction tips. Samples were injected to a Dionex Ultimate 3000 RSLCNano System (Thermo Fisher Scientific, Waltham, MA, USA) using a C18 trap-column (Dionex) and an in-house packed (3 µm C18 particles, Dr Maisch) analytical 50 cm x 75 µm emitter-column (New Objective, Littleton, MA, USA). Peptides were eluted at 200 nl/min with a 5-40% B 120 min gradient (buffer B: 80% acetonitrile + 0.1% formic acid, buffer A: 0.1% formic acid) to a Q Exactive Plus (Thermo Fisher Scientific, Waltham, MA, USA) mass spectrometer (MS) using a nano-electrospray source (spray voltage of 2.5 kV). The MS was operated with a top-10 data-dependent acquisition strategy. Briefly, one 350-1400 m/z MS scan at a resolution setting of R=70 000 at 200 m/z was followed by higher-energy collisional dissociation fragmentation (normalised collision energy of 26) of 10 most intense ions (z: +2 to +6) at R=17 500. MS and MS/MS ion target values were 3e6 and 5e4 with 50 ms injection times. Dynamic exclusion was limited to 40 s. The mass spectrometry proteomics data have been deposited to the ProteomeXchange Consortium via the PRIDE [11] partner repository with the dataset identifier PXD051211.

Raw mass spectrometric data files were processed using MaxQuant software and searched against *Saccharomyces* Genome Database proteins sequences of all systematically named ORFs as described previously [9, 12]. Detection of at least two peptides was taken as minimal number to consider corresponding protein as detected. Peptides originated from eL41 were not detected. Peptides originated from eL37, eL40, P1A/B, P2A/B, uL10, uL11, uL14, uL29 and uS19 were excluded from analysis due to extremely high variability or absence in one of biological replicates. The ‘heavy’/’light’ ratios were calculated as described previously [9]. For ribosomal proteins, fold change higher or lower than 1.41 times ($\log_2 >= 0.5$ or $\log_2 <= -0.5$) and *P*-value lower than 0.05 ($-\log_{10} > 1.3$) were defined as statistically significant thresholds of difference.

REFERENCES

1. Guldener U, Heck S, Fielder T, Beinhauer J, Hegemann JH. A new efficient gene disruption cassette for repeated use in budding yeast. Nucleic Acids Res. 1996;24(13):2519-24. doi: 10.1093/nar/24.13.2519.
2. Sikorski RS, Hieter P. A system of shuttle vectors and yeast host strains designed for efficient manipulation of DNA in Saccharomyces cerevisiae. Genetics. 1989;122(1):19-27. doi: 10.1093/genetics/122.1.19.
3. Harger JW, Dinman JD. An in vivo dual-luciferase assay system for studying translational recoding in the yeast Saccharomyces cerevisiae. RNA. 2003;9(8):1019-24. doi: 10.1261/rna.5930803.
4. Plant EP, Nguyen P, Russ JR, Pittman YR, Nguyen T, Quesinberry JT, et al. Differentiating between near- and non-cognate codons in Saccharomyces cerevisiae. PLOS ONE. 2007;2(6):e517. doi: 10.1371/journal.pone.0000517.
5. Maden BE. Mapping 2'-O-methyl groups in ribosomal RNA. Methods. 2001;25(3):374-82. doi: 10.1006/meth.2001.1250.
6. Leppik M, Peil L, Kipper K, Liiv A, Remme J. Substrate specificity of the pseudouridine synthase RluD in Escherichia coli. FEBS J. 2007;274(21):5759-66. doi: 10.1111/j.1742-4658.2007.06101.x.
7. Ero R, Leppik M, Liiv A, Remme J. Specificity and kinetics of 23S rRNA modification enzymes RlmH and RluD. RNA. 2010;16(11):2075-84. doi: 10.1261/rna.2234310.
8. O'Connor M, Leppik M, Remme J. Pseudouridine-Free Escherichia coli Ribosomes. J Bacteriol. 2018;200(4). doi: 10.1128/JB.00540-17.
9. Kisly I, Remme J, Tamm T. Ribosomal protein eL24, involved in two intersubunit bridges, stimulates translation initiation and elongation. Nucleic Acids Res. 2019;47(1):406-20. doi: 10.1093/nar/gky1083.
10. Tamm T, Kisly I, Remme J. Functional Interactions of Ribosomal Intersubunit Bridges in Saccharomyces cerevisiae. Genetics. 2019;213(4):1329-39. doi: 10.1534/genetics.119.302777.
11. Perez-Riverol Y, Bai J, Bandla C, Garcia-Seisdedos D, Hewapathirana S, Kamatchinathan S, et al. The PRIDE database resources in 2022: a hub for mass spectrometry-based proteomics evidences. Nucleic Acids Res. 2022;50(D1):D543-D52. Epub 2021/11/02. doi: 10.1093/nar/gkab1038.
12. Piir K, Tamm T, Kisly I, Tammsalu T, Remme J. Stepwise splitting of ribosomal proteins from yeast ribosomes by LiCl. PLOS ONE. 2014;9(7):e101561. doi: 10.1371/journal.pone.0101561.
13. Baxter-Roshek JL, Petrov AN, Dinman JD. Optimization of ribosome structure and function by rRNA base modification. PLOS ONE. 2007;2(1):e174. doi: 10.1371/journal.pone.0000174.
